# Geospatial optimization of Air-Mobile Stroke Unit deployment in Norway: expanding the frontiers of neurocritical care

**DOI:** 10.3389/fneur.2026.1740806

**Published:** 2026-03-09

**Authors:** Caroline J. Jagtenberg, Annemijn M. Boer, Marius Rehn, Jo Roislien, Maren R. Hov, Karianne Larsen

**Affiliations:** 1 Department of Operations Analytics, Vrije Universiteit Amsterdam, Amsterdam, Netherlands; 2 Department of Research and Development, Norwegian Air Ambulance Foundation, Oslo, Norway; 3 Institute of Clinical Medicine, University of Oslo, Oslo, Norway; 4 Division of Pre-Hospital Services, Oslo University Hospital, Oslo, Norway; 5 Faculty of Health Sciences, University of Stavanger, Stavanger, Norway; 6 Department of Neurology, Oslo University Hospital, Oslo, Norway; 7 Faculty of Health Sciences, Oslo Metropolitan University, Oslo, Norway

**Keywords:** acute stroke treatment, Air Mobile Stroke Unit, emergency medical services, geospatial analysis, Helicopter Emergency Medical Services

## Abstract

**Introduction:**

Rural and remote communities face disadvantages in acute stroke care. An Air-Mobile Stroke Unit (Air-MSU), adapting traditional MSUs for aircraft, could enable timely prehospital assessment in underserved regions. This modeling study aimed to identify the optimal Air-MSU base in Norway to maximize patient coverage, using increased geographic reach within 4.5 h as a proxy for clinical efficacy and improved outcomes.

**Materials and methods:**

All Helicopter Emergency Medical Services (HEMS) bases in Norway were evaluated as candidate sites using 2022 Norwegian Stroke Registry data at the postal-code level. Postal codes within a 15-min drive of a hospital were excluded to reflect realistic ground-ambulance coverage. Additional analyses focused on rural patients located more than 150 min from a hospital, assumed ineligible for hyperacute treatment within 4.5 h of onset. A Maximum Covering Location Problem (MCLP) model identified the HEMS base that maximized patient coverage within a 150-min response window.

**Results:**

Positioning the Air-MSU at Dombås in Central Norway covered 87.5% of stroke patients, increasing nationwide hyperacute treatment availability from 91.8 to 94.8%. For rural patients, the optimal base was Harstad in Northern Norway, covering 14.7% of all stroke patients but increasing the total proportion eligible for treatment within 4.5 h to 97.2%.

**Discussion:**

Locating an Air-MSU in Harstad would most effectively improve access to hyperacute stroke care in rural and remote Norway. Geospatial modeling combined with mathematical optimization supports strategic planning of future prehospital stroke services.

## Introduction

Rapid diagnostics and treatment of acute stroke is crucial for patient prognosis, and efforts should be made to improve the chain of stroke survival (1–4). Mobile stroke units (MSUs) are road ambulances equipped with a Computed Tomography (CT) scanner, a point-of-care laboratory and a specially trained stroke team (5, 6), and enable diagnostics and treatment of acute stroke. MSU care has been found to reduce time to treatment and improve patient functional outcomes (7, 8). MSUs render accurate prehospital stroke subtyping which opens for hyperacute treatment of both cerebral ischemia and hemorrhages. Accurate field-diagnosis also allow direct transfer to thrombectomy or neurosurgery. MSUs have been found to be effective for outcomes and costs, in densely populated areas with shorter driving distances (7, 9–12), and geospatial analyses have previously been performed to optimize patient coverage and treatment times in such urban settings (13–15). In contrast, evidence supporting MSU implementation in rural regions remains limited (16).

Countries with a widely dispersed population and large rural areas face differences in the care of stroke patients (17–20). Stroke patients residing in rural or remote areas have longer prehospital times, are less likely to receive acute stroke treatment, and have an increased stroke-related mortality (19, 20). Distance to the hospital alone may exclude these patients from effective acute treatment, and solutions to extend acute stroke care into these disadvantaged areas are needed. In Australia, with large differences in geography and population density, only 3% of individuals residing in rural areas have access to timely stroke care, in contrast to 77% of those living in urban areas (18).

Norway, with large variations in population density, long distances and challenging geography, faces similar challenges, and yearly stroke reports show considerable differences in reperfusion therapy rates between hospitals (17, 21). The Norwegian Ministry of Health and Care Services aims for equity of access to health care regardless of place of residence, and that 90% of the population should be reached by a physician-manned ambulance within 45 min (22, 23). This cannot be achieved with ground ambulances alone. Norway has 13 Helicopter Emergency Medical Services (HEMS) bases with 14 operative rotor-wing air ambulances staffed with an anesthesiologist, a rescuer and a pilot (24). HEMS is indispensable in countries with geographic barriers or low population, and benefits transport from rural areas to specialized care (25).

To address urban–rural disparities an Air-Mobile Stroke Unit (Air-MSU) has been proposed (26, 27). Air-MSU approach adapts the conventional road-based MSU model for deployment via fixed-wing or rotor-wing aircraft. Although CT technology does not yet offer devices compact enough to be transported by helicopter, ongoing developments of durable, compact scanners may enable practical testing of Air-MSU concept (26–28). To be suitable for medical air transport, such scanners must meet several aviation and medical standards. The device must be lightweight to minimize impact on aircraft range and be configured as a nonpermanent installation that operates on an external battery on the ground, ensuring flexibility and safety during both transport and clinical use. A clinical study on the feasibility of this new CT technology is expected in the near future (29).

There are several possible configurations for an Air-MSU, from equipping an existing rotor-wing aircraft with a CT scanner for stroke response, to establishing a dedicated, standalone unit.

In any case, a future Air-MSU will come at a cost, and it is imperative to optimally position such a service, to maximize its effect on the stroke population. Throughout this paper, increased geographic coverage is used as a meaningful proxy for clinical efficacy at population level —more patients reached within 4.5 h from symptom onset - indicating greater access to timely reperfusion and improved outcomes. We use models to identify the optimal HEMS base at which to locate such a hypothetical Air-MSU. We aimed to identify which of the existing Norwegian HEMS bases is the best location to deploy a future Air-MSU, and how many stroke cases it could serve if this innovative stroke service were to be introduced.

## Materials and methods

In this mathematical simulation study, we explored the optimal positioning of a future Air-MSU service in Norway in a series of numerical experiments, using historical stroke registry data from 2022. Throughout this paper we assume a setup consisting of a dedicated, standalone Air-MSU helicopter, that is placed in addition to the existing HEMS, thereby not interfering with non-stroke missions.

### Setting

We only consider the Norwegian total land area, except Svalbard archipelago, with an area of 323.810 km^2^ and a distance between its northern and southern end, 1750 km (30). In 2022, mainland Norway was subdivided into 357 municipalities and 3,368 distinct postal codes with a total population of 5.4 million (31, 32).

To reflect realistic ground ambulance coverage, we excluded patients residing in postal codes within a 15-min drive of any of the 52 Norwegian hospitals. We conducted one experiment (Experiment 1) for all stroke cases in the remaining postal codes and a second experiment (Experiment 1R) focused on patients located >150 min from a hospital – defined as *rural* stroke cases - assumed ineligible for hyperacute treatment within 4.5 h of stroke onset.

The HEMS response times were defined as the time between emergency call and arrival of the helicopter on scene. We calculated the service area of each of the 52 hospitals and the transport time for each of the 3,368 postal codes by processing data through Quantum Geographic Information System (QGIS), a free, open-source software that facilitates editing and visualization of geographic information (33). Finally, the optimal HEMS base from which to deploy an Air-MSU was identified by implementing the mathematical model described below.

### Data

#### Stroke data

We used historical stroke data from Norway in 2022, 8,938 acute strokes provided by the Norwegian Stroke Registry which is the national medical quality registry for acute stroke cases in Norway (34). It systematically collects data on patients aged ≥18 years admitted to Norwegian hospitals with acute stroke (ICD-10 codes I61, I63, I64), with the aim of monitoring and improving the quality of stroke care and outcomes. Reporting of acute stroke admissions to the registry by all hospitals that treat stroke is mandatory by law, and the registry has a stable coverage degree of above 85% when compared with administrative hospital data sources. Aggregated results are publicly available and used for quality improvement and research.

In the current study we made no distinction between ischemic and hemorrhagic strokes. And as data input to the mathematical optimization, we used how the number of strokes were divided over each of the 3,368 unique postal codes.

#### Transportation data

A map of Norway with 3,368 postal code polygons were obtained from GEONORGE [a] (35), for which geographical centroids were determined using QGIS. The municipality corresponding to each postal code was found in GEONORGE [b] (36).

The QGIS software allowed to create, analyze, and visualize geospatial information. The Norwegian road network (GEOFABRIK) (37) was used as a QGIS input layer. This distinguishes the different types of roads with their corresponding maximum allowed speeds, from 25 km/h to 100 km/h. Only roads accessible by car were selected. The few road links that did not have a maximum speed were manually filled with the average speed limit of the corresponding road type.

**Figure 1:**
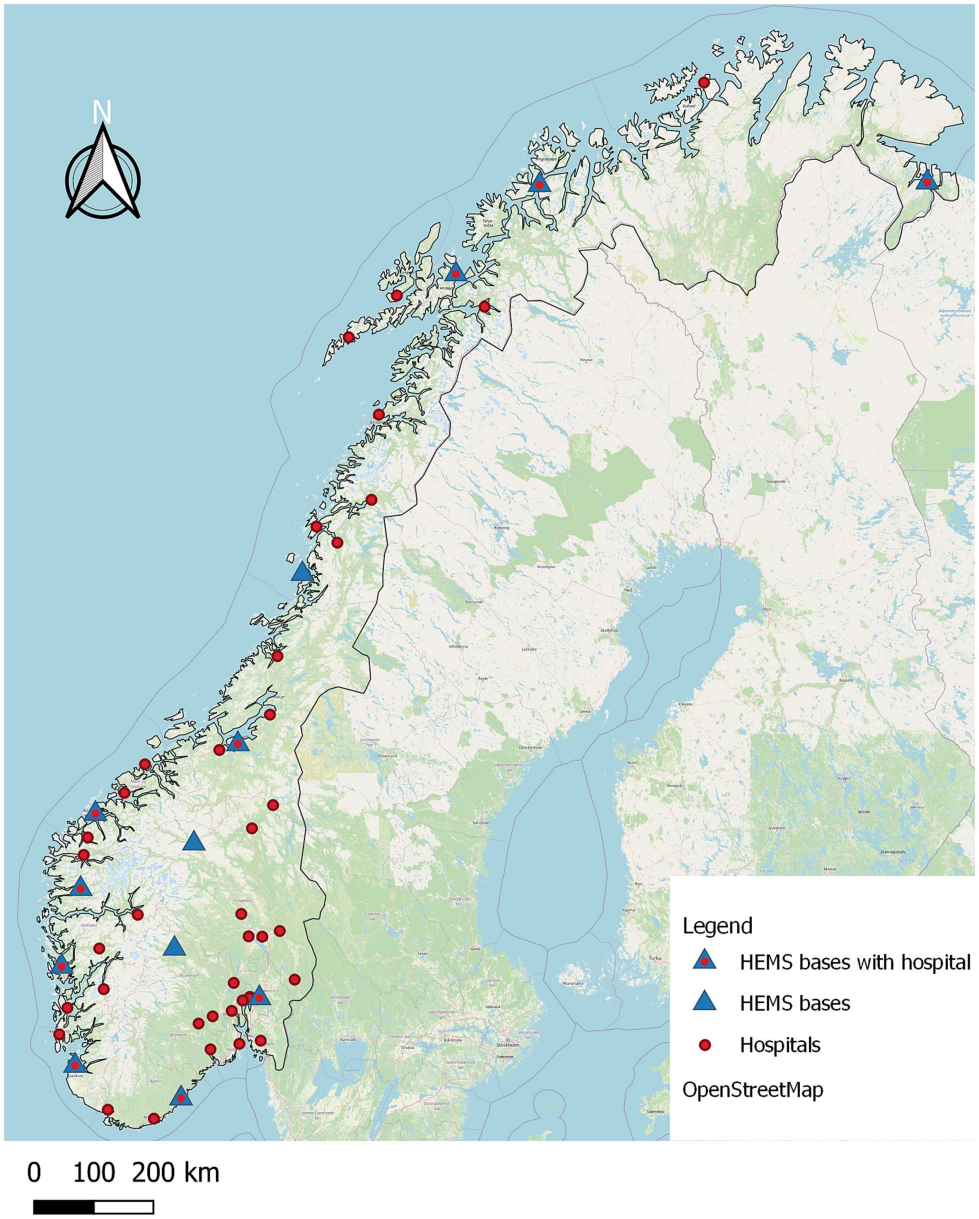
The distribution of all 52 hospitals and 13 HEMS bases across Norway. The centroids of some of the hospitals overlap, which results in less than 52 being visible on the map. Data: ©OpenStreetMap contributors, ODbL 1.0.

The coordinates of the 52 acute hospitals and 13 HEMS bases in Norway were obtained using Google Maps (Google, USA) and imported to QGIS as vector layers with centroids representing the facilities. An acute hospital was defined as a hospital providing emergency medical care with on-site CT capability for acute stroke diagnostics; the corresponding postal codes were obtained from information provided by the four Regional Health Authorities. Hospital locations and HEMS bases are shown in Figure 1. We used QGIS to identify which postal codes were a > 150-min drive from a hospital.

For each postal code, we calculated whether stroke patients could reach the hospital in time for hyperacute stroke treatment under two predefined care pathways: (1) conventional hospital-based treatment and (2) Air-MSU-based treatment. The hyperacute treatment window was set at 4.5 h (270 min) from symptom onset, corresponding to the standard treatment window for tissue Plasminogen Activator (tPA) (38). A decision time of 90 min from symptom onset to first medical contact was assumed, based on the reported median decision time in a Norwegian ischemic stroke population (39). For Pathway 1 (conventional hospital-based treatment) a door-to-needle time of 30 min was assumed, approximating the national mean in 2022 (17). Under this pathway, the maximum allowable time for prehospital transport (including ambulance response and travel time) was therefore calculated as 270–90 – 30 = 150 min. For Pathway 2 (Air-MSU-based treatment), the time from helicopter arrival to thrombolytic treatment (helicopter door-to-needle)was also set to 30 min, based on previous MSU studies and the assumption that the Air-MSU workup and new CT technology would not add additional time compared to conventional road-based MSU workflows (40, 41). Under this pathway, the maximum allowable prehospital transport time was likewise 150 min. Thus, for both pathways, a threshold of 150 min was applied for total prehospital time from medical transport to treatment unit (emergency response + transport). Ambulance response time was assumed 12 min in municipalities >10.000 inhabitants and 25 min elsewhere, per national recommendations (42). The remaining time required to transport the patient to the hospital, the transport time, was estimated using the OD-matrix function in QGIS. This function generates the driving time in seconds, using the fastest path between the centroid of the patient’s postal code and the closest hospital, assuming all roads are travelled at their respective speed limit. The total time (sum of ambulance response time and transport time) for each postal code is stored in an OD-matrix. The equipment is permanently installed so that jobs received while in-flight do not incur a forced return to the base for installment.

For some postal codes, calculating the transport time in the manner described above yielded no result, typically because the centroid did not lie along any road. In such cases, we artificially connected the centroid to the nearest existing road, with a maximum speed of 60 km/h. Finally, some of the postal codes are islands, which a ground ambulance can realistically reach by ferry, but this information is not contained in the road network data. To represent such a ferry ride, we added dummy roads following the shipping lines of ferries with a maximum speed of 15 km/h. Using this combined road network, we derived which postal codes can reach the nearest hospital by ground ambulance within 150 min. These patients are theoretically eligible for hyperacute treatment within 4.5 h, even without Air-MSU.

Air-MSU response time consists of preflight preparation time and actual flying time. The distance between postal code centroids and all HEMS bases was calculated in QGIS. Subsequently, as used in earlier literature, an average speed of 220 km/h plus 5.5 min of preflight preparation time were used to estimate the duration of a flight between any base and any postal code (43, 44).

### Mathematical model

The optimal location for an Air-MSU was calculated using the Maximal Covering Location Problem (MCLP) (45). MCLP is a model that finds the location(s) from which most patients are reachable within a given range, in our case: 150 min. The two previously described experiments (Experiment 1 and Experiment 1R) were conducted using the MCLP model with inputs according to the selection criteria.

All open data sources are listed in the Supplementary material; additional data are available on request.

### Ethics

This project used no health related or identifiable data and did not require ethical approval. Data was provided by open web sources, and the anonymous stroke count was provided by the Norwegian Stroke Registry. Informed consent was not applicable as no personal information or data were used in this research.

## Results

### Data analysis

**Figure 2:**
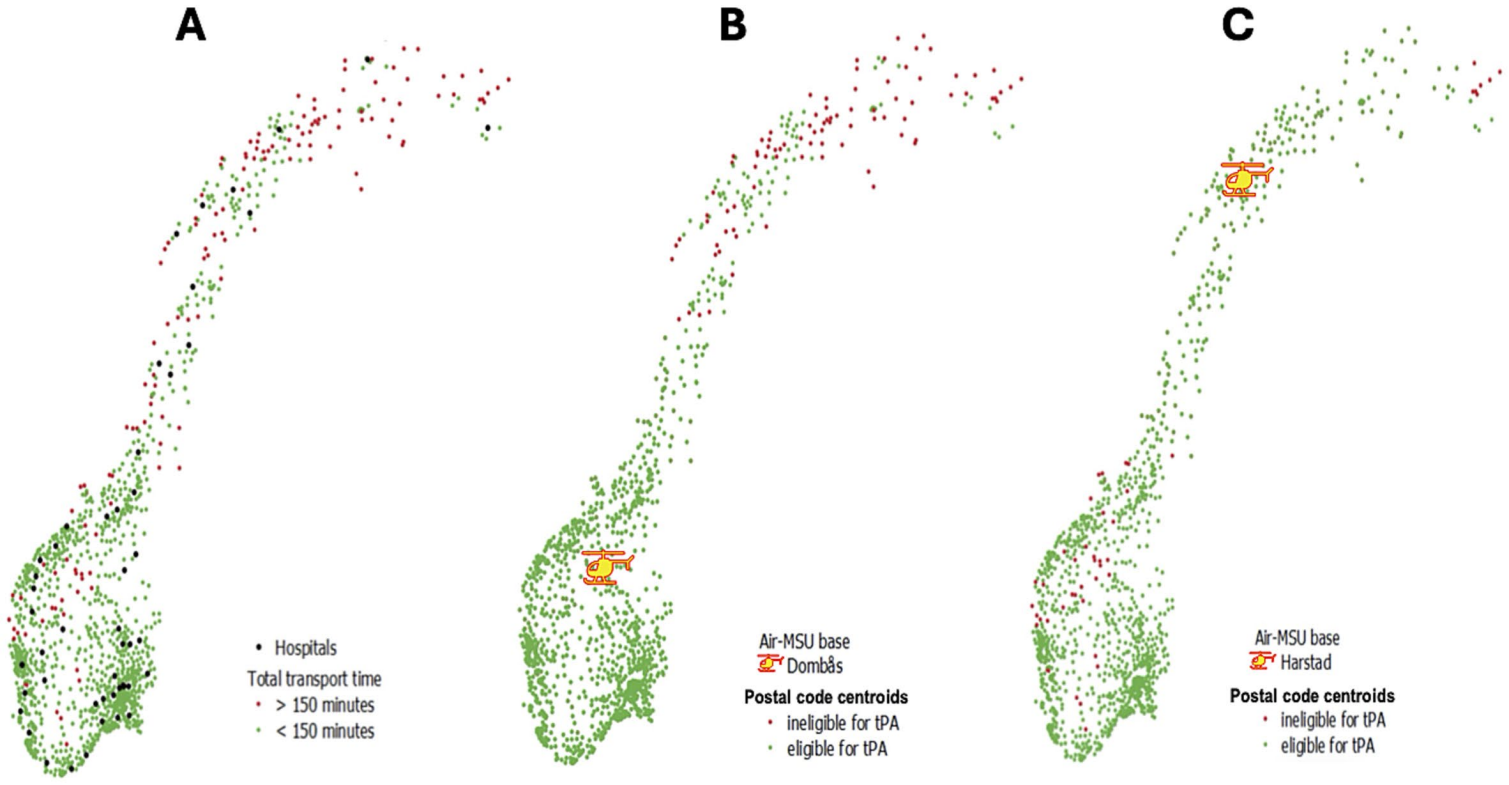
Maps of Norway illustrating stroke populations and results from the experiments. **(A)** Locations where patients are eligible for acute treatment within 4.5 h are shown in green (≤150-min drive to hospital) and ineligible locations in red (>150-min drive to hospital). **(B)** Air-MSU base in Dombås: green dots indicate postal codes that are theoretically reachable within 4.5 h by Air-MSU or by road ambulance to hospital (94.8%). Patients in these postal codes can be transferred to hospital within 150 min by ground ambulance or are covered by the Air-MSU (Exp. 1). **(C)** Air-MSU in Harstad: Green dots indicate postal codes that are theoretically reachable by Air-MSU or by road ambulance to hospital within 150 min (Exp. 1R). Air-MSU, Air-Mobile Stroke Unit; tPA, tissue Plasminogen Activator; Exp, experiment.

Of the total 3,368 postal codes in Norway, 1,437 were within 15 min of a hospital, and excluded from the study. From the remaining 1931 postal codes, there were 1,286 in which one or more patients suffered a stroke in 2022. In those 1931 postal codes combined, there were 4,375 strokes. Summary statistics for the surface area and stroke count per postal codes are provided in the Supplementary Table 1. Upon calculating the transport time for all postal codes, 142 (11%) required manual editing. In total, 141 postal codes (11%) where 360 stroke cases resided in 2022 (8.2% of the total stroke cases that year) were estimated to have ground transportation times to hospital exceeding 150 min (Figure 2A). That means– without Air-MSU and relying on ground transport only - the remaining (4375–360)/4,375 = 91.8% of strokes are already able to reach a hospital within 150 min; this is our baseline. The mathematical model yielded two experiments (Table 1).

**Table 1:** Summary of MCLP experiments and results.

Exp.	Study population	Postal codes (*n*)	Cases^2^ (*n*)	Optimal location	Coverage by Air-MSU (%)	Total stroke coverage <4.5 h^4^ (%)	Response time (min)
Main analysis: 150-min response time							
1	Strokes^1^	1,286	4,375	Dombås	87.5	94.8	96.9
1R	Rural strokes^1^	141	360	Harstad	65.6/14.7^3^	97.2	242.6^3^

The experiments marked with R (rural) are performed for the 141 rural postal codes which exceed a total transport time of 150 min to hospital. ^1^Stroke count for the year 2022. ^2^Excluding patients closer than 15 min driving time from a hospital. ^3^Of all stroke patients (*n* = 4,375) in 2022. ^4^Stroke patients reached by Air-MSU or road ambulance to hospital. Main analysis was performed with an Air-MSU response time of 150 min. Exp., Experiment; Air-MSU, Air-Mobile Stroke Unit; min, minutes; hrs, hours; n, number.

### MCLP

The optimal HEMS base to position Air-MSU according to the MCLP model was computed in a series of experiments, each with varying parameters and input data (Table 1).

#### Coverage with a 150-min response time

**Figure 3:**
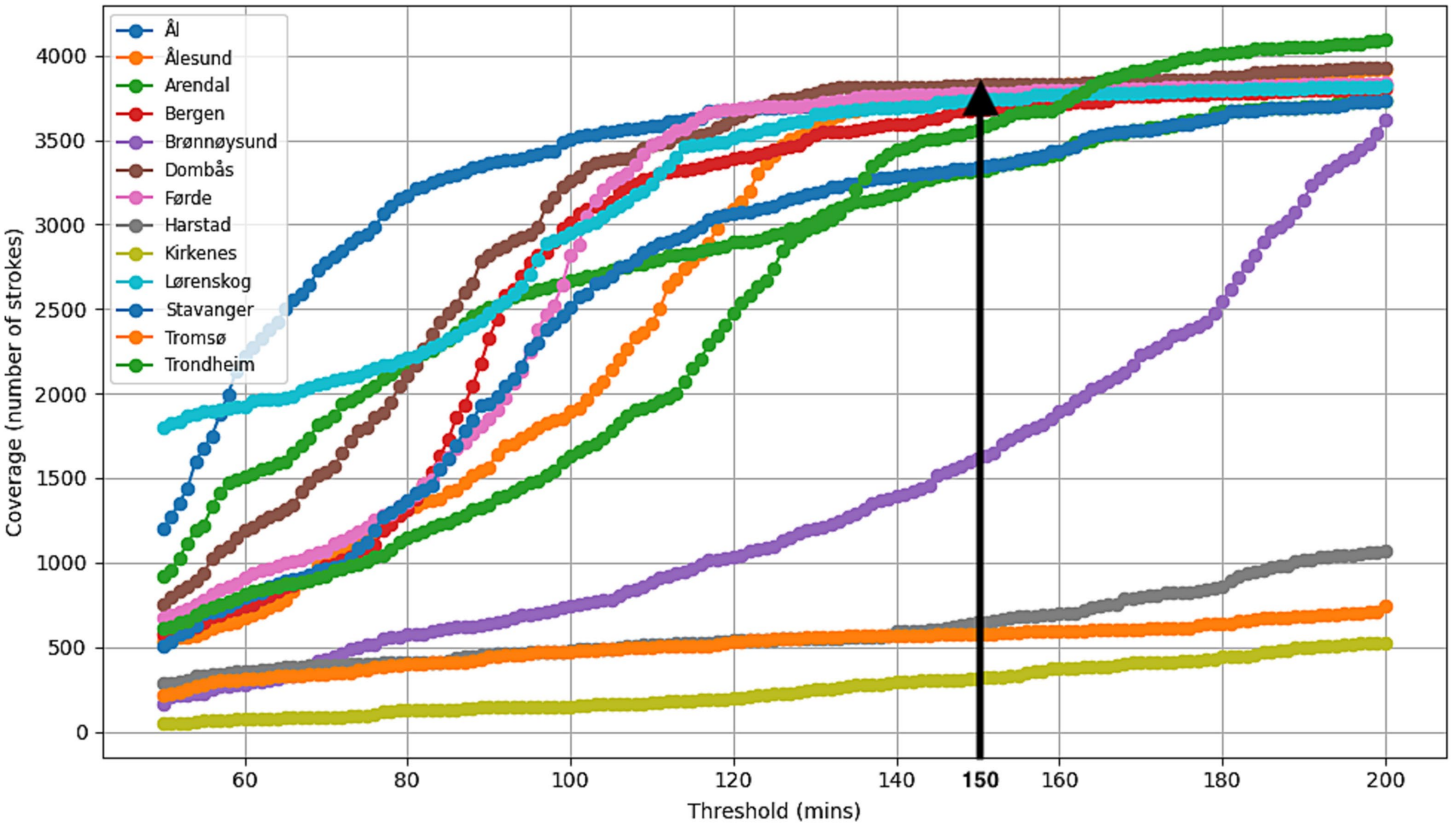
The figure illustrates how stroke coverage and the optimal base location evolve with increasing response time, showing that Dombås is the optimal location for a response time of 150 min.

All strokes (Experiment 1): Considering the 13 existing HEMS bases with 150 min response time, optimal stroke coverage was achieved when positioning the Air-MSU at the HEMS base in Dombås, in Central Norway. This covered 3,826 stroke cases (87.5%), with average response time of 96.9 min to all 4,375 cases in 1286 postal codes. The effect of placing the Air-MSU at Dombås can be viewed as follows: Of the 141 postal codes that have a total transport time of >150 min, 53 would be covered by Air-MSU from Dombås: an expected 131 verified stroke cases per year (3%). In total, 4,146 stroke cases (94.8%) can be transported to a hospital by ground ambulance or be reached by Air-MSU in 150 min (Table 1, Figure 2B). Figure 3 illustrates how stroke coverage and the optimal base location vary as a function of response time.

#### Coverage of rural areas with a 150-min response time

**Figure 4:**
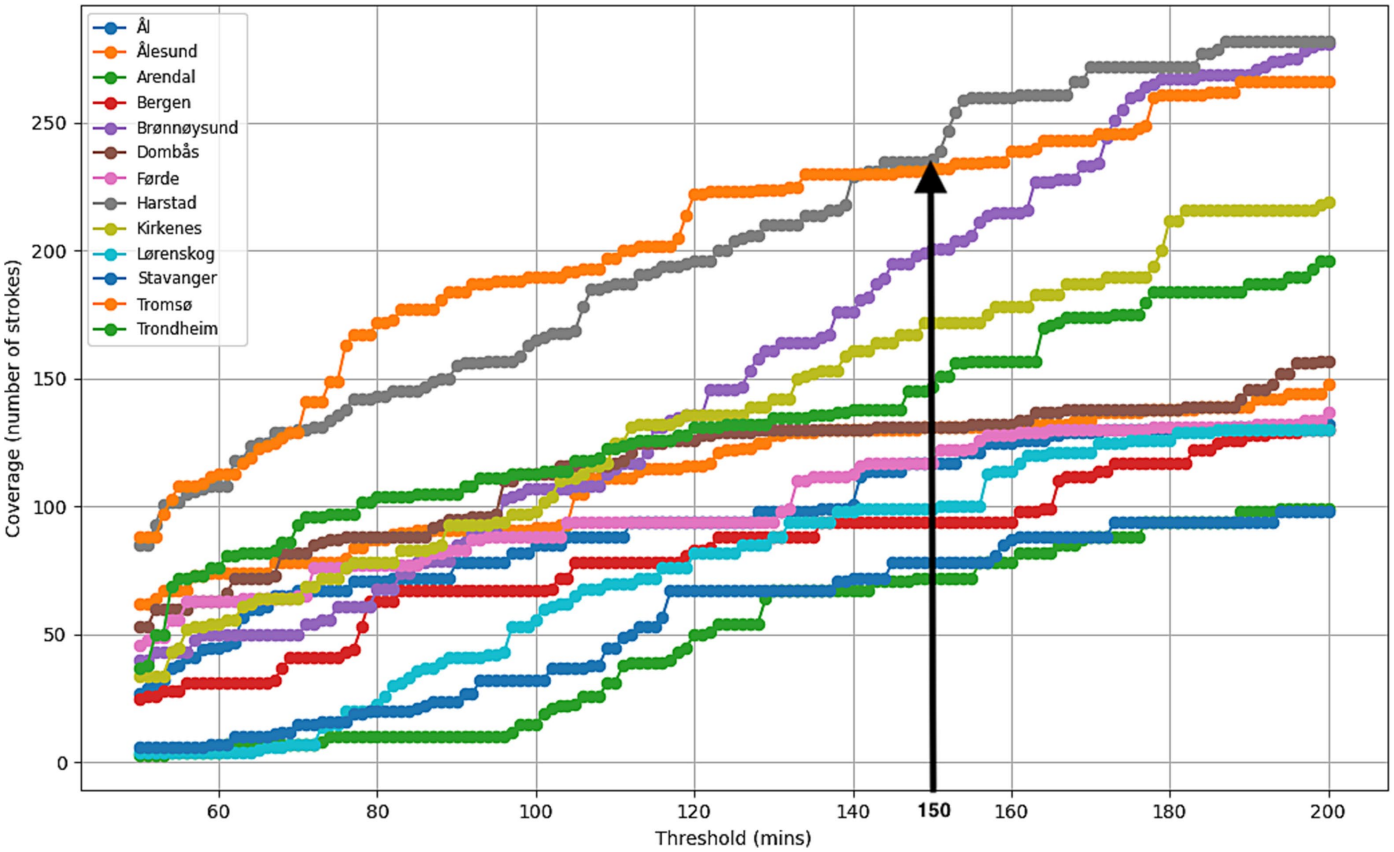
The figure illustrates how rural stroke coverage and the optimal base location evolve with increasing response time, showing that Harstad is the optimal location for rural stroke coverage for a response time of 150 min.

Rural strokes (Experiment 1R): Focusing on rural patients, maximal stroke coverage was achieved by placing Air-MSU at HEMS base in Harstad in the Northern Norway. A total of 236 (65.6%) of the 360 rural stroke cases would then be covered by Air-MSU and eligible for acute stroke treatment. The average response time to the patients would be 242.6 min. Placing Air-MSU in Harstad would cover only 644 (14.7%) of all 4,375 historical stroke cases as compared to the 87.5% when locating it at the Dombås base suggested from Experiment 1. However, in Harstad it would increase the total of all cases theoretically being eligible for acute treatment within 4.5 h from 91.8 to 97.2% (Figure 2C). Figure 4 illustrates how rural stroke coverage and the optimal base location vary as a function of response time.

## Discussion

This study presents an innovative national, geospatial simulation integrating air and road prehospital networks, and is the first to identify which existing air ambulance base locations would provide optimal Air-MSU coverage. The findings can inform decision-makers on strategic location of future Air-MSUs in Norway, and the methodology can be adapted to identify optimal Air-MSU locations in other countries or regions. Formulating the problem as an MCLP and using the Gurobi solver allows for optimal deciding on the locations of multiple Air-MSUs simultaneously; however, the single-location problem can also be solved in a simpler way by computing the coverage for each of the 13 HEMS bases and selecting the one with the highest coverage.

The study shows that an Air-MSU service may improve the availability of hyperacute stroke care in the population and expand stroke treatment to rural areas without access to acute stroke care. More specifically, with a 150-min response time, Air-MSU optimally covers the annual total stroke population when it is based in Dombås. The analysis also demonstrates that optimal Air-MSU base location is dependent on the response time threshold applied. Conversely, Harstad in Northern Norway would be the optimal location considering hyperacute stroke care coverage in rural areas with large distances to hospital excluding timely stroke treatment. Placing the Air-MSU in Harstad, also leads to the highest availability of hyperacute stroke treatment for the nationwide stroke population (97%).

Postal codes in remote and rural areas represent patients with an increased risk of missing acute stroke treatment. Expanding prehospital stroke care with an Air-MSU can enable timely stroke treatment also for these disadvantaged patients. It also contributes to the health political goal of equal access of health care for all. Research has shown that stroke patients living in rural areas have less access to acute stroke care, and worse outcomes than patients living in urban areas with easy access to high quality acute stroke care (18–21). Air-MSU complements the ground ambulance and may expand the number of patients who are eligible for tPA treatment by reducing the time to diagnostics and treatment for patients in rural areas. This study has demonstrated that to optimize coverage of rural patients unable to reach the hospital in time for acute treatment, Air-MSU must be located further north compared to the optimal location for general patient coverage. To maximize patient coverage and reduce treatment times for more patients within 150 min, our advice is to locate Air-MSU in Dombås. However, we encourage practitioners to reconsider the trade-off between efficiency and increasing the total number of patients. With a focus on the *additional* patients that can receive treatment, the Harstad base would be considered as the best alternative. There is a north to south paradox in Norwegian stroke care, where the Norwegian Stroke Registry’s yearly reports indicate a higher stroke rate in Northern areas, but also fewer hospitals and longer distances to acute stroke care (34). This may suggest that more stroke patients in these areas get delayed treatment and worse prognosis (21).

Whether Air-MSU should operate as a dedicated stroke response unit or integrate into the existing air ambulance services remains uncertain. Determining the most effective model will require clinical trials exploring the efficacy and cost-effectiveness of Air-MSU deployment. The optimal configuration will likely vary based on regional differences in geography and population demographics.

Today, HEMS has variable and typically few dispatches to acute stroke patients (46), possibly due to HEMS not having the diagnostic tools to subtype acute strokes, leading to no available acute stroke treatment, other than symptomatic and life-saving interventions. With access to advanced prehospital diagnostics HEMS could become a crucial part of the stroke care chain of survival. Research has also indicated that including acute stroke in HEMS, even without treatment options, could improve HEMS cost-effectiveness (46). A previous study also suggests that any strategies to improve the thrombolytic rate, even those requiring significant investments, will most likely result in cost savings as the investments could quickly be repaid by the reduced dependency of treated patients (2).

We defined hyperacute stroke treatment as the 4.5-h thrombolytic window, noting that this phase after symptom onset is likewise critical for management of cerebral hemorrhages (47). Air-MSUs will be able to subtype strokes enabling hyperacute, field-treatment both for ischemic and hemorrhagic strokes. The decisive effect of timely thrombolytic treatment in ischemic stroke is well-established (1), but recent studies have also underlined the beneficial effect of early treatment in hemorrhagic strokes (48–50). Anesthesiologists are trained to deliver neuro-protective care. Prehospital treatment of acute stroke in road MSUs has shown to be timesaving, outcome-improving and safe (7, 8). A Norwegian MSU trial proved timesaving and safety of an MSU staffing model aligned with the Norwegian nationwide HEMS, wherein an anesthesiologist serves as the onboard MSU physician (41). The HEMS anesthesiologists are specifically trained to perform advanced prehospital medical procedures including neuroprotective interventions - an essential component of effective Air-MSU operations. The integration of advanced acute stroke diagnostics and treatment of acute stroke into the existing scope of HEMS crews are enabled through an Air-MSU and based on previous MSU research, a feasible, logical and safe alternative to moving in-hospital stroke specialists into HEMS. Nonetheless, staffing of Air-MSU must be adapted to local conditions, and optimal diagnostic accuracy and therapeutic decision-making will heavily depend on remote consultation with stroke specialists and neuroradiologists and the implantation of artificial intelligence-based diagnostic decision support.

Even though an Air-MSU would introduce a different prehospital setting than the road MSU, many aspects are similar. The primary clinical and radiological diagnostics and start of acute treatment will be performed on ground also with an Air-MSU, but a smaller workspace, air transport and off-road locations introduce new challenges that need to be addressed. Also, the austere prehospital HEMS unit environment will challenge a future Air-MSU service. The diagnostic device must comply with applicable aviation and medical certification requirements, emphasizing the need for new and operationally durable CT technology (28).

Direct field triage of selected patients to comprehensive stroke centers for thrombectomy and neurosurgery will also be enabled with Air-MSU as already demonstrated in road MSU studies (7, 41, 51–53). Our results suggest that Air-MSUs are especially important in extending acute treatment to populations in rural and remote areas, and since the distance to few and centrally located comprehensive stroke centers are vast in these setting, Air-MSU can introduce considerable time reductions to thrombectomy and neurosurgery. Given the long, shared border between Norway and Sweden, vast distances and HEMŚ long range capability, cross-border mutual-aid agreements for acute stroke response may also be relevant in sparsely populated regions, and similar approaches have previously been explored in European settings (54). In an Air-MSU model it will be unlikely to have an on-board neurologist and radiologist/radiographer mainly due to costs and the logistic and practical challenges associated with in-hospital personnel working in an austere prehospital environment. Telemedical support like teleradiology and videoconference with in-hospital specialists can be a proper solution in settings with acceptable mobile network coverage as shown in other prehospital studies (55–57). Training prehospital air-ambulance personnel to staff the Air-MSU is also a solution, and this setup has been proven safe and time effective earlier in a Norwegian road MSU study (41). The study was inspired by the national HEMS system, and equipped the MSU similar to the standard rotor-wing air ambulance, and staffed the MSU with a specially trained 3-person team, similar to that in HEMS; an anesthesiologist (air-ambulance physician), a paramedic (pilot) and paramedic-nurse (rescuer). The anesthesiologist is specially trained to handle prehospital medical and traumatic emergencies, and acute stroke would be added to the work list. In a future perspective, artificial intelligence in clinical examination, new stroke diagnostic tools, and blood biomarkers could play an important role in the prehospital diagnostics of acute stroke (29, 58, 59), but today traditional radiological imaging is mandatory before starting stroke treatment.

In this study, we observed a higher stroke admission rate in the middle and northern regions of Norway, consistent with data reported in several annual reports of the Norwegian Stroke Registry (34). The reasons for this apparent south–north gradient in stroke incidence remain unclear, and warrant further investigation.

Air-MSU may be a future solution to increase availability of acute stroke care, although significant challenges remain regarding its implementation. Experiences with ground MSU and ambulances show very low accuracy in stroke dispatches (41, 60, 61), meaning that more than half of the stroke dispatches are for conditions other than stroke. Current evidence indicate that this affects cost-effectiveness of MSUs, ground or air, as MSUs are proven effective for confirmed stroke patients, with uncertain benefit for non-stroke patients (7, 11, 62, 63). An Air-MSU depends on solid prehospital clinical diagnostics and effective logistics throughout the prehospital stroke care chain of survival. Tools to improve prehospital accuracy of stroke diagnosis would include training and increased competence of prehospital personnel and digital tools for effective transfer of information and data. This could also include decision support by artificial intelligence and videoconferencing with stroke specialists. Stroke severity and distance to hospital would be among several aspects that have to be considered before dispatching an Air-MSU. Placing a CT scanner in a helicopter will not automatically solve the problem of geographical inequalities in acute stroke care, as this heavily relies on optimized health systems starting with patient awareness and accurate stroke dispatch. Only in these settings can a specialized unit like the Air-MSU fulfill its potential.

### Limitations

The model assumes that Air-MSU would serve large geographic and demographic areas serving several health regions and overlapping with national HEMS. This setup could work in a study, but to be integrated into HEMS it is probably more realistic that the CT scanner is on-loaded whenever dispatched to a stroke patient, meaning that the scanner would be a non-permanent installation like the incubator for newborns. We did not account for the potential added time for on-loading a mobile CT scanner into the helicopter. Traditional CT technology has proved not feasible to be used in helicopters; however, the final setup, specifications and approvals for CT scanners or other innovative stroke diagnostic devices in aircrafts are not decided, which is a major limitation to this study (28, 29). As it was not in the scope of this article, we did not consider the logistic and economic consequences of how this setting would change the dispatch panorama for HEMS, including the challenge of receiving in-flight dispatches.

The utilization of QGIS enabled the identification of the state according to transport time for the 1,286 Norwegian postal codes. The existing road network and speed limits reliable estimates of total ground transport times. We did not consider the effect of blue light driving on transport times (64), and did not include models for rendezvous between Air-MSU and ground ambulance, which in reality is sometimes necessary. The model only evaluated postal codes with at least one stroke occurrence in 2022, ignoring postal codes with zero strokes. Our study used strict limits for the time interval between the onset of symptoms and the emergency call and the door-to-needle time, while in real life these numbers are subject to considerable variation.

The MCLP model inexplicitly assumes the Air-MSU would be available whenever needed, ignoring challenges in weather or concurrency conflicts. Ignoring the latter is supported by a previous study on concurrency conflicts in Norwegian HEMS (65). Norwegian HEMS already fly to patients with high pre-test probability stroke in cases where ground transport will infer a significant delay to tPA/thrombectomy. However, adding more stroke dispatches to the worklist would likely introduce new concurrency conflicts. The model did not consider incorrect stroke dispatches (stroke mimics) which today is a challenge for prehospital stroke services (66).

We only considered the effect of Air-MSU on stroke patient coverage within hyperacute phase. Other medical conditions may benefit from CT scanners in HEMS, like traumatic brain injuries, but these effects were not considered in this paper.

In conclusion, an Air-MSU service can improve the availability of hyperacute stroke care and expand acute treatment to rural areas without access to acute stroke care. Our results show that the Air-MSU covers the highest number of stroke cases when placed in Dombås, where it would cover 87.5% of the stroke population. Combined with ground ambulances, this would result in a total of 94.8% of strokes available for treatment within a 4.5-h time window. When focusing on patients living in rural areas excluding them from hyperacute stroke care, placing the Air-MSU more North, in Harstad, would be most effective. In this position it optimally complements ground transport resulting in 97.2% of the nationwide stroke population being available for acute stroke treatment within the hyperacute time window. Geospatial analyses can aid decision making on Air-MSU location with the goal of optimizing prehospital acute stroke care.

## Data Availability

The data analyzed in this study is subject to the following licenses/restrictions: the datasets were from both publically available sources and upon request from the Norwegian Stroke Registry - where the total amount of confirmed strokes are publically available however anonymized stroke count per postal code were provided by the Norwegian Stroke registry. All open data sources are listed in the Supplementary material; additional data are available on request. Requests to access these datasets should be directed to karianne.larsen@norskluftambulanse.no and for stroke registry data request through: https://www.stolav.no/fag-og-forskning/medisinske-kvalitetsregistre/norsk-hjerneslagregister.
