# Supplementary material for: Geospatial optimization of Air-Mobile Stroke Unit deployment in Norway: expanding the frontiers of neurocritical care
Source: Front Neurol. 2026 Mar 9;17:1740806. doi: 10.3389/fneur.2026.1740806 (PMC13006308; doi:10.3389/fneur.2026.1740806)
Supplement: Supplementary file 1 [file Table_1.docx]

## SUPPLEMENTAL MATERIAL

Supplemental Table 1:

| Postal code surface area (km^2^) | All of Norway^1^ | Experiment 1 | Experiment 1R |
| --- | --- | --- | --- |
| mean | 128.5 | 215.2 | 534.5 |
| median | 25.1 | 102.8 | 324.9 |
| 10^th^ percentile | 0.3 | 5.3 | 50.9 |
| 90^th^ percentile | 344.2 | 528.0 | 1434.3 |
| Stroke cases (n) | 8938 | 4375 | 360 |
| mean | 3.6 | 3.4 | 2.6 |
| median | 3.0 | 2.0 | 2.0 |
| 10th percentile | 1.0 | 1.0 | 1.0 |
| 90th percentile | 7.0 | 7.0 | 5.0 |

Summary statistics for the surface area and stroke count per postal code in 2022.

^1^excluding the Svalbard archipelago.

**Data sources**

All datasets with associated sources used during QGIS analyses

| **QGIS layer** | **Source** | **Information** |
| --- | --- | --- |
| OpenStreetMap | QGIS | EPSG: 3857 |
| Zip codes | GEONORGE [a] | Zip code areas in EPSG:25833 in format GeoJSON.  Change CRS using ”vector layer save as” in QGIS into EPSG:3857 |
| Municipality | GEONORGE [b] | Kommuner 2024 (to have the most updated information).  Format GEOJSON |
| Roads | GEOFABRIK | Norway; gis osm roads free 1.shp in EPSG:4258.  Change CRS using ”vector layer save as” in QGIS into EPSG:3857 |
| HEMS bases | Luftambulansetjenesten | Coordinates obtained with Google Maps (WGS 84, EPSG:4326).  Transformed using GmbH to coordinate system EPSG:3857 Final document name: Helicopter bases flight times |
| Hospitals | The Norwegian Air Ambulance Foundation | Coordinated obtained with Google Maps (WGS 84, EPSG:4326).  Reproject to EPSG:3857 using ”vector layer save as” in QGIS Final document name: Hospital coordinates all EPSG |
| Strokes | Norwegian Stroke Registry | Number of strokes per zip code in 2022 |

## References

Gurobi Optimization, LLC. (n.d.). Gurobi optimizer. https://www.gurobi.com/
